# Developing and validating a machine learning pharmaceutical therapy recommender system for US-based hospital in-patients with schizophrenia spectrum disorders

**DOI:** 10.1186/s12888-025-07657-8

**Published:** 2025-12-01

**Authors:** Maximin Lange, Urvik Mehta, Nikolaos Koutsouleris, Feras Fayez, Ricardo Twumasi

**Affiliations:** 1 Institute of Psychiatry, Psychology & Neuroscience, King’s College London, 16 De Crispingy Park, SE5, London, UK; 2 Barts and the London School of Medicine and Dentistry, London, UK; 3 Queen Mary, University of London, London, UK; 4 Ludwig Maximilian University, Munich, Germany; 5 Max Planck Institute of Psychiatry, Munich, Germany; 6 Imperial College Healthcare NHS Trust, London, UK

**Keywords:** Schizophrenia, Machine learning, Medication recommendation, Collaborative filtering, Antipsychotics, Clinical decision support, Personalised medicine, Treatment outcomes

## Abstract

**Background:**

No drug recommender system exists to guide antipsychotic medication selection for hospital in-patients with schizophrenia. This study developed and validated a prototype personalised medication recommender system for in-hospital patients with schizophrenia.

**Methods:**

This prognostic study analysed data from the Medical Information Mart for Intensive Care IV database on patients prescribed antipsychotic medications with hospital admission diagnoses of schizophrenic disorders between 2008 and 2022. Five similarity-based algorithms (two collaborative filtering and three distance-based representation methods) were evaluated using nested patient-wise cross-validation. The best performing model underwent external validation across geographic and temporal contexts using (a) Northwestern Intensive Care Unit and (b) Medical Information Mart for Intensive Care III datasets. Treatment success was measured through an affinity score (range 0–1) incorporating time between admissions, medication switches, and length of stay. Algorithm performance was assessed using root-mean-square error and mean average precision at position three, with visit-specific analysis across first, second, and third admissions.

**Results:**

Among 1,152 patients (660 first visits, 251 s visits, 241 third visits), cosine similarity-based collaborative filtering with K = 7 neighbours achieved optimal performance. Internal cross-validation demonstrated excellent prediction accuracy (root-mean-square error 0.116, standard deviation 0.007) and moderate recommendation quality (mean average precision at position three 0.490, standard deviation 0.029), improving to 0.697 (standard deviation 0.019) for first visits only. Geographic validation on 32 patients maintained recommendation quality (mean average precision at position three 0.432) despite increased prediction uncertainty (root-mean-square error 0.498). Temporal validation on 66 patients showed similar patterns (mean average precision at position three 0.359, root-mean-square error 0.578). Second-visit recommendations demonstrated exceptional transferability across validation contexts, achieving mean average precision at position three scores up to 0.813. In 163 cases where the model disagreed with attending clinicians, clinicians’ top-3 recommendations achieved significantly superior treatment outcomes (mean difference − 0.122 affinity points, 95% confidence interval: -0.145 to -0.098, *p* < 0.001, Cohen’s d=-0.81).

**Conclusions:**

This proof-of-concept study demonstrates feasibility of developing a machine learning-based medication recommender system for hospital in-patients with schizophrenia, that appear to maintain recommendation quality across different healthcare institutions and time periods. Prospective clinical trials are needed to establish real-world effectiveness and safety.

**Supplementary Information:**

The online version contains supplementary material available at 10.1186/s12888-025-07657-8.

## Background

Antipsychotic medications are the cornerstone and first-line option for treatment, effectively relieving some symptoms of psychosis [1, 2]. Even though all common antipsychotics outperform placebo [3], response rates are volatile, with sometimes almost half of patients not responding to first line treatment [4]. Thus, finding an effective antipsychotic often remains a process of trial and error [5].

Consequently, assistance is needed regarding the choice of antipsychotic medication, ideally bespoke to each patient.

Artificial Intelligence can offer guidance in healthcare when multiple potential baseline factors are available that could predict an outcome [6, 7]. Individual predictors of antipsychotic treatment response have successfully been identified, and attempts to anticipate treatment outcome have been conducted, as have general illness prognosis prediction (For reviews, see: [8, 9]). Reviews highlight a great potential for the use of AI in these tasks but lament the need for more precise and reliable predictions, as palpable benefits remain inconsistent.

In general medicine, machine learning based healthcare recommender systems have gained prominence (For reviews, see: [10–12]). A subset of healthcare recommender systems are drug or treatment recommender systems [12–15]. While there are multiple ways of approaching treatment recommender systems [16], we focus on collaborative filtering. Collaborative filtering (CF) algorithms, a form of machine learning, have been successfully used in e-commerce and entertainment [17–19]. These algorithms share basic concepts with medication recommendations. In both, items are being ranked based on individual predictors. Medications can thus be treated like items in an e-commerce environment, and clinical efficacy can be treated as past purchase behaviour. The most appropriate treatment with respect to a predefined outcome can be recommended to clinicians, who can share this with patients [15].

No treatment recommender system for schizophrenia exists today. Efforts in these directions have been made by Wu and colleagues [20], who developed and validated an individual treatment rule (ITR) for first episode schizophrenia patients in a large Taiwanese sample. However, their definition of treatment success was binary as not having switched medication or having been readmitted to hospital for any cause within one year.

In this study we develop a machine learning tool using sociodemographic and clinical variables of the MIMIC-IV database to recommend pharmaceutical treatment options relevant for medium-term outcomes of individual hospital patients experiencing schizophrenia. We then validate this model’s performance using NWICU and MIMIC III datasets.

We emphasise that our work is a proof-of-concept demonstration rather than a definitive prescribing tool; the model does not incorporate important clinical nuances such as formulation, dosage, or side-effect burden, and is intended to highlight feasibility and stimulate further research rather than guide clinical practice at this stage. We further acknowledge that schizophrenia care is marked by sociodemographic disparities, often over-diagnosing and under-treating minorities [21, 22], which the present study is not designed to resolve. Future work will be needed to evaluate whether such models can help mitigate, rather than reinforce, existing disparities.

## Methods

### Dataset for model development

For model development, we used MIMIC-IV [23] v. 3.1, a publicly available database containing deidentified electronic health records from over 360,000 individual patients admitted to the Beth Israel Deaconess Medical Center (BIDMC) in Boston, MA, USA, between 2008 and 2022.

### Patients

For inclusion, patients had to meet the following criteria: aged 18–40 years; ICD 9 or 10 diagnosis of schizophrenic, schizophreniform, or schizoaffective disorder (ICD9 295.X or ICD10 F2X).

### Data cleaning and preprocessing

When multiple ICD codes were present in comma-separated format, only the first code was retained for analysis. ICD-9 codes were systematically mapped to their ICD-10 equivalents. Specifically, schizoaffective disorder codes (295.7x) were mapped to F25.9, various schizophrenia subtypes were mapped to their corresponding F20.x codes (e.g., paranoid schizophrenia 295.3x → F20.0, disorganised schizophrenia 295.1x/295.2x → F20.1), and other psychiatric diagnostic codes were similarly converted to maintain consistency with current diagnostic standards. Full mapping is to be found in the code at [https://github.com/maximinl/scz_med_recommender].

Ethnicity variables were consolidated into broader categories to ensure adequate sample sizes for analysis. White subcategories (including Russian, Eastern European, and other European designations) were grouped as “White.” Black/African American categories (including Caribbean and Cape Verdean) were consolidated into “Black/African American.” Hispanic/Latino subcategories (Cuban, Puerto Rican, Dominican, Central American, Mexican, etc.) and South American designations were grouped as “Hispanic/Latino.” Asian subcategories were consolidated into “Asian.” Records classified as Unknown/Declined/Other, American Indian/Alaska Native, Native Hawaiian/Other Pacific Islander, or Multiracial were excluded from the analysis due to small sample sizes.

Four patients who had documented second visit dates but lacked initial visit records were excluded to maintain data integrity. This was likely due to wrong entry at the source i.e. the hospital at data collection.

### Treatment outcome

*Note: the following section provides the full mathematical specification of the affinity score. While technical details may be skipped by readers less interested in equations*,* the underlying components (readmission*,* length of stay*,* visits*,* and medication switches) are central to interpreting the clinical relevance of our findings. In simple terms*,* the affinity score combines four routine clinical signals (how soon patients are readmitted*,* how often they are hospitalised*,* how long they stay in hospital*,* and whether they need to switch medications) into a single measure of how well a treatment worked*,* with a penalty applied when patients switch to a second or third antipsychotic*,* and a bonus when no switch occurs.*

Outcome for each prescribed antipsychotic is measured by an affinity score, defined between 0 (poor outcome) and 1 (good outcome). The score is calculated by averaging four normalised components: (1) total time between recent admissions, (2) total length of hospital stay, (3) number of prior visits, and (4) time between medication switches. These are scaled to the $\:\left[\text{0,1}\right]$range using population-level min–max normalisation. A penalty is subtracted if the patient switched to a second or third antipsychotic after initiating the treatment in question. The final score is clipped to $\:\left[\text{0,1}\right]$. We limit the computation to the first three visits and up to two switches to reduce runtime.

For each patient–drug instance we compute min–max–normalised components oriented so that larger = better:

- $t$: time between admissions (higher → fewer relapses).
- $s$: time between medication switches (higher → greater stability).
- $\ell$: 1 – norm(length of stay)
- $v$: 1 – norm(number of visits)

With training-fold statistics for normalisation, the base score is the equal-weight average:

$$\:b=\frac{1}{4}\left(t+s+\mathcal{l}+v\right)$$

We then apply a fixed penalty scheme, with a small bonus for no switch and penalties for first or second switches, and clip the final score to [0,1].

$$\:A=\text{clip}(b-P,\text{\hspace{0.17em}}0,\text{\hspace{0.17em}}1),P=\{\begin{array}{cc}-0.05&\:\text{no\:switch\:(bonus)}\\\:0.10&\:\text{first\:switch}\\\:0.20&\:\text{second\:switch}\end{array}$$

We emphasise again the proof-of-concept nature of this work: our goal was to test how well a simple and transparent measure could perform the recommendation task, rather than proposing a definitive clinical outcome score. Our approach also follows the precedent of Gräßer [15], who employed an analogous equal-weighted composite outcome in a therapeutic recommender framework and whom we follow closely in this work. Similarly, choice of penalty was heuristic and deliberately simple, following again Gräßer who applied a fixed − 0.25 penalty for adverse events in their affinity score. Our goal was not to identify an optimal penalty scheme but to establish a proof-of-concept framework that could be refined in future work.

#### Sensitivity analysis

To assess robustness of model performance under different affinity score composition, we conducted sensitivity analyses, redefining the affinity score with alternative weighting schemes and penalty magnitudes: In addition to the original equal-weight formulation, we implemented a stability-heavy variant (prioritising longer intervals between admissions and fewer medication switches) and a patient-burden–heavy variant (prioritising fewer hospital visits and shorter length-of-stay, with reduced penalties for switching).

### Antipsychotic medications

Antipsychotic drugs included in this study were aripiprazole, asenapine, chlorpromazine, clozapine, fluphenazine, haloperidol, iloperidone, olanzapine, paliperidone, perphenazine, prochlorperazine, risperidone, thiothixene, ziprasidone, haloperidol, lurasidone. These were all the antipsychotics that were prescribed for our selected patient cohort in the MIMIC-IV database. We also included the option of no medication as a treatment option.

### Predictors

Patient demographic and clinical characteristics, including gender, race, and age, were identified from the records. In addition, information was extracted about medication transitions (time between first to second and second to third medication switches), hospital utilisation patterns (including length of stay across three visits and time intervals between admissions), and comorbidity burden (calculated as a count of concurrent conditions).

### Algorithms

We implemented five similarity-based algorithms: two collaborative filtering (CF) approaches and three distance-based representation (DR) methods. The CF approaches used cosine similarity and Euclidean distance converted to similarity scores using similarity = 1 / (1 + distance + ε), where ε = 1e-9 prevents division by zero. The DR methods employed: (1) Gower similarity coefficient combined with Relief-Based Attribute weighting using ReliefF for feature selection (DR-RBA), (2) Gaussian Radial Basis Function transformations with exponential kernels (DR-RBF), and (3) Neighborhood Component Analysis for feature space optimisation (DR-NCA). The DR-RBA and DR-NCA methods use a threshold of 0.5 to create binary labels for Relief-based feature selection and neighborhood component analysis, respectively. All algorithms further use this same threshold (0.5) to distinguish between good and poor outcomes when calculating the MAP@3 evaluation metric, in concordance with previous works [15].

## Model performance evaluation

### Hyperparameter optimisation

We employed a nested patient-based cross-validation strategy to avoid data leakage due to consultations from the same patient. The outer loop used 5-fold cross-validation, randomly assigning approximately 20% of patients to each test fold, while training the model on the remaining 80%. Within each outer fold, hyperparameter optimisation specifically targeted the neighbourhood size (K). We used an inner 5-fold GroupKFold cross-validation on the training data, again ensuring that consultations from the same patient remained within the same inner fold. Candidate K values ranged from 5 to 60 in increments of 5, and the optimal value was selected based on minimizing the root-mean-square error within the inner validation loop.

### Visit based evaluation

Strict measures were implemented to prevent data leakage throughout the evaluation process. We implemented a comprehensive visit-based evaluation framework that respects the temporal nature of clinical decision-making. For each patient visit, the model uses only information that would have been available to clinicians at that specific time point. For Visit 1, the model uses only training data from other patients. For Visit 2, the model uses training data plus the patient’s previous visit (Visit 1). For Visit 3, the model uses training data plus the patient’s previous visits (Visits 1–2). This approach prevents data leakage while providing realistic performance estimates for clinical deployment scenarios.

**Fig. 1:**
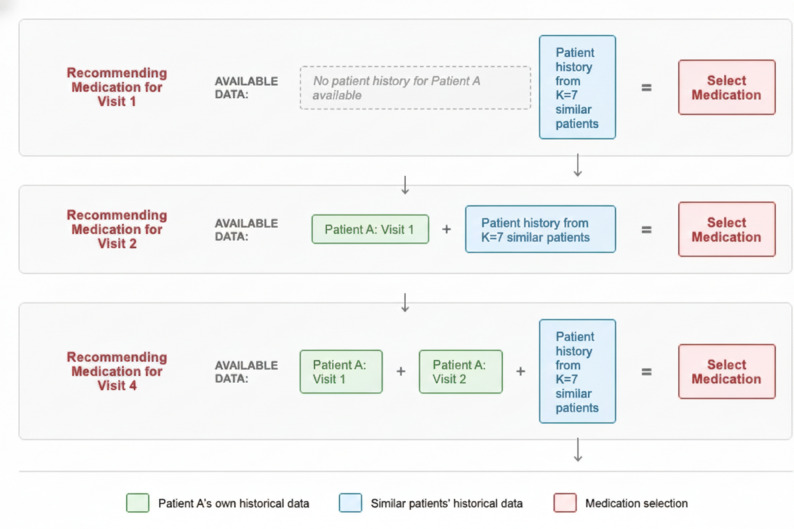
Visit based evaluation

Affinity score calculation for the test set used only training set statistics for normalisation bounds, ensuring that no information from future consultations influenced past predictions. Feature preprocessing components including one-hot encoders and standard scalers were fitted exclusively on training data and subsequently applied to test data. Temporal constraints within the visit-based evaluation ensured only temporally appropriate information was used for each prediction. For a visualisation see Fig. 1.

### Evaluation metrics

Root-Mean-Square Error (RMSE) measured prediction accuracy between predicted and observed affinity scores. Mean Average Precision at 3 (MAP@3) evaluated recommendation quality by assessing whether effective treatments (affinity score > 0.5) appeared in the top 3 recommendations. Overlap quantified the proportion of test cases where the actual prescribed therapy appeared in the top 3 recommendations. Coverage represented the proportion of test cases where the actual prescribed therapy did not appear in the top 3 recommendations (1 - Overlap).

### Clinical decision comparison analysis

For cases where the best performing model recommendations disagreed with clinician prescribing decisions (actual therapy not in top 3 recommendations), we analysed the difference between predicted affinity scores for the AI’s top recommendation versus the actual achieved affinity score of the clinician’s choice. This analysis provided insight into scenarios where clinical judgment may capture factors not represented in the available data features, particularly examining the magnitude and direction of predicted improvement when the model disagreed with clinical decision-making.

### External validation

#### Datasets

For external validation, we employed MIMIC-III v1.4 [24] and Northwestern ICU (NWICU) v.0.1.0 [25].

##### MIMIC III

MIMIC III is an independent patient cohort from the same hospital as MIMIC-IV but covering an earlier time period of 2001–2012. MIMIC-IV and III have some initial overlap, however, when selecting only *CareVue* patients [26], these are not included in MIMIC IV, and hence can be used for validation. The same inclusion criteria were applied to MIMIC-III as described for the development dataset. Patient data SQL extraction code can be found at [https://github.com/maximinl/scz_med_recommender].

##### NWICU

NWICU is a publicly available database containing deidentified electronic health records from over 25,000 individual patients admitted to Northwestern Memorial HealthCare in Chicago, IL, USA, between 2020 and 2021. The clinical cohort included all patients admitted to an ICU and subsequently discharged in 2020 and 2021, regardless of COVID-19 status. The same inclusion criteria were applied to NWICU as described for the development dataset. Patient data python extraction code can be found at [https://github.com/maximinl/scz_med_recommender].

##### Best performing model

The final model with optimal hyperparameters was trained on the aforementioned MIMIC-IV patients and subsequently evaluated on MIMIC-III and NWICU patients without any further parameter tuning or model modification. This external validation approach tests model generalisability across different time periods and clinical practice patterns while maintaining complete independence between development and validation datasets. The same evaluation metrics and visit-based framework were applied to the external validation to ensure consistent performance assessment. Results are reported separately for Visit 1, Visit 2, Visit 3, and aggregated across all visits.

## Results

### Patients included (Model development)

We screened 364,627 unique patients for study eligibility between 2008, and 2022. As described above, admission records were capped at the 3rd visit.

After all cleaning procedures, the final dataset comprised 1,152 unique patients contributing 1,885 total visits (Table 1). The mean age was 27.9 ± 6.4 years. Most patients were male (65.8%) and of White ethnicity (44.4%), with approximately two-fifths being Black (39.6%). Over half of patients (57.3%) had only a single recorded visit during the study period. Over half of all patients (55.8%) did not receive any antipsychotic medication during their hospital stays. Among those who did receive antipsychotics (44.2% of patients), the most prescribed medications were risperidone (17.4% of all patients), olanzapine (17.0%), and haloperidol (15.1%). Aripiprazole was prescribed to 9.6% of patients, while clozapine was used in 4.9% of cases. The remaining 2.6% of patients received other antipsychotic medications not among the five most prescribed agents. There were substantial differences in sample sizes across visit types, with Visit 1 consultations comprising the majority of evaluations (377.8 ± 13.06 samples on average) compared to Visit 2 (99.0 ± 9.65 samples) and Visit 3 (48.4 ± 4.84 samples).

**Table 1:** Patient cohort

Characteristic		*N*
Total patients		1152
Total Visits		1885
Age (SD)	27.9 ± 6.4	1152
Length of Stay (SD)	6.6 ± 10.5	1885
**Gender**		
M	65.8%	758
F	34.2%	394
**Ethnicity**		
White	44.4%	512
Black	39.6%	456
Hispanic/Latino	8.1%	93
Other	7.9%	91
**Number of Antipsychotics**		
None	22.4%	258
1	14.1%	163
2	6.6%	76
3	1.0%	11
4	0.1%	1
5	22.4%	258
**Antipsychotics Prescribed**		
None	55.8%	643
Risperidone	17.4%	201
Olanzapine	17.0%	196
Haloperidol	15.1%	174
Aripiprazole	9.6%	111
Clozapine	4.9%	56
Others	2.6%	30
**Number of Visits**		
1	57.3%	660
2	21.8%	251
3	20.9%	241

### Model selection

The visit-based cross-validation results demonstrate distinct performance patterns across different similarity methods and visit types (Table 2). All methods showed consistent hyperparameter selection, with optimal K values ranging from 6.0 to 8.0 across different approaches.

#### Collaborative filtering approaches

CF (Cosine) achieved an overall RMSE of 0.118 (± 0.004) with particularly strong performance on Visit 1 consultations (RMSE: 0.117 ± 0.007, MAP@3: 0.702 ± 0.019). The method maintained robust therapeutic outcome prediction with high overlap rates ranging from 0.662 (± 0.051) for Visit 2 to 0.874 (± 0.023) for Visit 1.

CF (Euclidean) showed comparable performance with an overall RMSE of 0.117 (± 0.004) and similar MAP@3 performance across visit types. Both collaborative filtering approaches demonstrated superior performance on first visits compared to subsequent visits, with Visit 1 achieving MAP@3 scores above 0.69 while Visit 2 showed more modest performance in the 0.23–0.30 range. Visit 3 had excellent RMSE of 0.094 (± 0.008), but atrociously low MAP@3 (below 0.02).

#### Distance-based representation methods

DR-RBA (Gower) achieved an overall RMSE of 0.119 (± 0.007) with consistent K selection at 7.0 (± 2.45) across all evaluations. The method demonstrated stable performance across visit types with MAP@3 scores closely matching those of CF approaches.

DR (Euclidean RBF) required slightly larger neighbourhoods with K = 8.0 (± 4.00) but achieved similar overall RMSE performance of 0.119 (± 0.005). The method showed consistent patterns across visit types, with Visit 1 performance (MAP@3: 0.696 ± 0.016) comparable to other methods but with slightly lower performance on subsequent visits.

DR-NCA (Euclidean) matched the performance of other distance-based methods with an overall RMSE of 0.119 (± 0.007) and optimal K = 7.0 (± 2.45). The method demonstrated consistent recommendation quality across visit types, with performance patterns similar to DR-RBA (Gower).

#### Visit-specific performance patterns

A consistent pattern emerged across all methods showing superior performance on first visits compared to subsequent consultations. Visit 1 evaluations achieved the highest MAP@3 scores (ranging from 0.696 to 0.702 across methods).

Visit 2 performance showed notable degradation across all methods, with MAP@3 scores dropping to the 0.23–0.30 range.

Visit 3 evaluations showed the most limited performance, with MAP@3 scores consistently below 0.02 across all methods.

**Table 2:** Visit-based cross-validation results

Algorithm	Best K	RMSE	MAP@3	Coverage	Overlap	Avg Samples
*CF (Cosine)*	7.0 (4.00)	0.118 (0.004)	0.493 (0.021)	0.186 (0.020)	0.814 (0.020)	377.8 (13.06)
Visit 1	7.0 (4.00)	0.117 (0.007)	**0.702 (0.019)**	0.126 (0.023)	0.874 (0.023)	230.4 (1.36)
Visit 2	7.0 (4.00)	0.138 (0.013)	0.297 (0.056)	0.339 (0.051)	0.662 (0.051)	99.0 (9.65)
Visit 3	7.0 (4.00)	**0.094 (0.008)**	0.012 (0.010)	0.157 (0.040)	0.844 (0.040)	48.4 (4.84)
*CF (Euclidean)*	6.0 (2.00)	0.117 (0.004)	0.491 (0.021)	0.189 (0.017)	0.811 (0.017)	377.8 (13.06)
Visit 1	6.0 (2.00)	0.114 (0.005)	0.699 (0.017)	0.135 (0.019)	0.866 (0.019)	230.4 (1.36)
Visit 2	6.0 (2.00)	0.138 (0.015)	0.238 (0.059)	0.336 (0.057)	0.664 (0.057)	99.0 (9.65)
Visit 3	6.0 (2.00)	0.097 (0.003)	0.012 (0.010)	0.149 (0.040)	0.851 (0.040)	48.4 (4.84)
*DR-RBA (Gower)*	7.0 (2.45)	0.119 (0.007)	0.493 (0.022)	0.186 (0.017)	0.814 (0.017)	377.8 (13.06)
Visit 1	7.0 (2.45)	0.118 (0.010)	0.701 (0.015)	0.129 (0.011)	0.871 (0.011)	230.4 (1.36)
Visit 2	7.0 (2.45)	0.140 (0.015)	0.239 (0.060)	0.330 (0.063)	0.670 (0.063)	99.0 (9.65)
Visit 3	7.0 (2.45)	0.097 (0.003)	0.012 (0.010)	0.158 (0.047)	0.842 (0.047)	48.4 (4.84)
*DR (Euclidean RBF)*	8.0 (4.00)	0.119 (0.005)	0.490 (0.023)	0.183 (0.017)	0.817 (0.017)	377.8 (13.06)
Visit 1	8.0 (4.00)	0.117 (0.006)	0.696 (0.016)	0.131 (0.014)	0.869 (0.014)	230.4 (1.36)
Visit 2	8.0 (4.00)	0.138 (0.014)	0.242 (0.059)	0.323 (0.064)	0.678 (0.064)	99.0 (9.65)
Visit 3	8.0 (4.00)	0.096 (0.007)	0.012 (0.010)	0.147 (0.060)	0.853 (0.060)	48.4 (4.84)
*DR-NCA (Euclidean)*	7.0 (2.45)	0.119 (0.007)	0.493 (0.022)	0.186 (0.017)	0.814 (0.017)	377.8 (13.06)
Visit 1	7.0 (2.45)	0.118 (0.010)	0.701 (0.015)	0.129 (0.011)	0.871 (0.011)	230.4 (1.36)
Visit 2	7.0 (2.45)	0.140 (0.015)	0.239 (0.060)	0.330 (0.063)	0.670 (0.063)	99.0 (9.65)
Visit 3	7.0 (2.45)	0.097 (0.003)	0.012 (0.010)	0.158 (0.047)	0.842 (0.047)	48.4 (4.84)

Mean and standard deviation of outcome prediction accuracy (RMSE), recommendation list agreement (MAP@3), average *coverage* of treatment options and *overlap* with applied treatment across visit-based cross-validation folds. CF = Collaborative Filtering, DR = Dimensionality Reduction, RBA = Relief-Based Attribute weighting, NCA = Neighborhood Components Analysis, RBF = Radial Basis Function. Bold values indicate best performance for RMSE (lowest) and MAP@3 (highest)

#### Sensitivity analyses

As shown in Supplementary Table 1, overall performance metrics remained broadly stable across different affinity score formulations. Averaged across visits, RMSE ranged from 0.118 to 0.139, overlap remained ~ 0.81. While MAP@3 values shifted modestly between variants, the relative performance profile was consistent.

#### Clinical decision comparison analysis

We analysed 163 Visit 1 cases where the CF (Cosine) model’s top 3 recommendations did not include the clinician’s prescribed therapy. In these disagreement cases, the system’s top recommendation achieved a predicted affinity score that was, on average, 0.12 points lower than the physician’s actual achieved affinity score (system: 0.45 ± 0.18 vs. physician: 0.57 ± 0.13, difference: -0.12 ± 0.15). A paired t-test confirmed this difference was statistically significant (t = -10.3653, *p* < 0.0001) with a large effect size (Cohen’s d = -0.8119).

#### External validation performance

Geographic validation on 32 patients from NWICU showed maintained recommendation quality (MAP@3 0.432) despite increased prediction uncertainty (RMSE 0.498). Temporal validation on MIMIC-III achieved predictive performance of MAP@3 of 0.359 and RMSE of 0.578 with Visit 1 consultations showing moderate performance (MAP@3: 0.298) and Visit 2 improving predictive accuracy (MAP@3: 0.813; Table 3).

**Table 3:** Results across different datasets

Dataset	Samples	RMSE	MAP@3
MIMIC-IV (Internal Validation)	1152	0.1155	0.4901
**Visit 1**	**660**	**0.1135**	**0.6965**
Visit 2	251	0.1351	0.2363
Visit 3	241	0.0928	0.0120
MIMIC-NW (External Validation)	32	0.4977	0.4323
Visit 1	24	0.4717	0.3750
Visit 2	6	0.4653	0.5833
Visit 3	2	0.6924	0.6667
MIMIC-III (Temporal Validation)	66	0.5775	0.3586
Visit 1	56	0.5803	0.2976
Visit 2	8	0.5193	0.8125
Visit 3	2	0.9249	0.2500

Note. RMSE = Root Mean Square Error; MAP@3 = Mean Average Precision at 3

## Discussion

We developed and validated a collaborative filtering-based medication recommender system for schizophrenia spectrum disorders using the MIMIC-IV dataset, with external validation on MIMIC-III and NWICU. Our primary model, CF (Cosine) with K = 7, demonstrated robust performance across datasets in predicting treatment outcomes, as measured by our composite affinity score, particularly for initial patient visits.

There was a strong alignment between our model’s recommendations and therapies prescribed by clinicians that led to good outcomes, especially for Visit 1 (MAP@3: 0.697 ± 0.019; Overlap: 0.874 ± 0.023). This concordance suggests that the engineered affinity score, which incorporates proxies for treatment success as readmission, length of stay, visit frequency, and medication stability, captures elements that are indeed indicative of positive treatment trajectories and align with clinical judgment. The model’s ability to predict these affinity scores with a low RMSE (0.117 ± 0.007 for Visit 1) supports the consistency of the patterns it learned. Furthermore, our sensitivity analyses suggest that our model’s performance is not dependent on a single arbitrary formulation of the affinity score but are robust to reasonable alternative definitions. The performance degradation observed for subsequent visits highlights the increasing complexity of treatment decisions as patients progress through their care journey.

In 163 instances where the model’s top 3 recommendations did not include the prescribed medication, clinicians achieved consistently better outcomes. This might indicate that variables available from our EHR datasets cannot capture the full breadth of clinical reasoning, including patient-specific side-effect considerations, prior treatment experiences, and contextual behavioural observations, or that our affinity score is missing crucial components. Identifying contexts where the model diverges from clinicians might be beneficial for guiding targeted model improvements.

## Limitations

The affinity score, while multifaceted, remains a proxy for true clinical success and may not capture all dimensions critical to patient well-being, such as specific side-effect profiles or individual patient preferences not documented in EHR. The definition of a “good outcome” using a threshold of 0.5 for the affinity score in MAP@3 calculations, although based on previous work, is a simplification. Observational data and unmeasured confounding variables may have influenced outcomes, and the explicit rationale behind clinicians’ prescribing decisions is not available.

While external validation was performed, both MIMIC IV and III datasets originate from the same institution, and NWICU is still within the US healthcare system. This limits generalisability to different healthcare systems, for example in Europe, with varied demographic populations or distinct clinical practice norms. Furthermore, all study datasets are derived from tertiary teaching hospitals and primarily from ICU settings, not a mental health hospital subset of patients, nor a psychiatric specialist service. Prescribing patterns may differ substantially from those in specialist psychiatric wards e.g., high haloperidol prevalence might be of a sedative nature rather than for its antipsychotic properties. Reliance on primary diagnosis codes may further introduce selection bias.

Our evaluation compares the model against the aggregate performance of many clinicians, and individual clinician expertise or variability is not assessed. The necessary consolidation of ethnicity categories and the exclusion of groups with small sample sizes for analytical stability mean that the model’s performance and applicability for these specific populations remain undetermined; future work must prioritise ensuring fairness and equitable performance across all demographic groups.

In external validation, performance decreased compared with internal testing, which highlights the model’s limitations and the effects of dataset shift between hospitals and restricts conclusions about later-stage treatment optimisation. Increased error should be interpreted as reduced calibration and generalisability, not as an adaptive property of the model. We further do not differentiate between antipsychotic medication as long acting injectables and oral formulations, nor do we respect dosage. Our model furthermore does not account for specific comorbidities, focusing only on the total comorbidity count. Our model learned from historical prescribing patterns in MIMIC-IV and thus may perpetuate societal and clinical biases rather than correct them, essentially encoding and perpetuating discriminatory practices. We deliberately employed collaborative filtering in line with prior work as a transparent, tractable baseline recommender; however, CF estimates correlations in observed outcomes rather than causal treatment effects, leaving it vulnerable to treatment-selection bias and unmeasured confounding.

## Future research directions

Refining the affinity score through direct clinician feedback and the incorporation of patient-reported outcomes are key next steps. Further investigation into the specific clinical factors driving model-physician disagreements could yield deeper insights into complex decision-making. Methodological advancements to improve predictive performance for subsequent visits are warranted. An important next step will be to enhance the interpretability of the recommender’s outputs. Although similarity-based collaborative filtering is more transparent than deep learning approaches, the rationale behind individual recommendations may not be obvious to clinicians. In practice, recommendations could be accompanied by (i) lists of the most similar historical patients and their treatment outcomes, (ii) a breakdown of how different affinity score components contributed to the ranking, and (iii) confidence indicators reflecting the density of similar cases in the training data. Such strategies would allow clinicians to situate model suggestions within their own reasoning process and may help integrate the system as a supportive rather than prescriptive tool. Extending and validating models in diverse healthcare settings and across broader patient demographics, with a specific focus on ensuring equitable performance for underrepresented groups, will be crucial for wider applicability. Here, cross-national validation is needed, with datasets from different continents, countries, and health care systems. Our limitation of not distinguishing long-acting injectable antipsychotics from oral agents could be countered via structured prescription data, which are available in more detailed EHRs or pharmacy records. Additionally, many EHR already feature prescription dosages which should be utilised. If not available, standardised daily dose equivalents (e.g., chlorpromazine equivalents) could be used to capture intensity of exposure. Side-effect burden might be assessed through adverse event codes, laboratory values (e.g., metabolic parameters, QT prolongation), and patient-reported outcome measures where available.

This study serves as a proof of concept, demonstrating that machine learning methods can be effectively adapted to psychiatric care to enhance clinical decision-making. Future research should aim to validate these findings in mental health specialist service cohorts. Additionally, embedding this approach into clinical workflows will require evaluation of its usability and trustworthiness from the perspective of both clinicians and patients.

## Conclusion

Our findings suggest that machine learning models operating on readily available pre-treatment data can offer individualised treatment trajectories for schizophrenia. By integrating structured clinical data, patient demographics, and outcomes from similar patients, this prototype demonstrates the feasibility of a scalable and interpretable therapy recommender system. However, realizing its full potential will require further validation in diverse settings and prospective studies to ensure its reliability and relevance in real-world care. This study effectively demonstrates that ML-based medication recommendation for schizophrenia is feasible and worth pursuing further.

## Supplementary Information

Below is the link to the electronic supplementary material.

Supplementary Material 1

Supplementary Material 2

## Data Availability

Source Code for all analysis reported can be found at: (https://github.com/maximinl/scz_med_recommender). In this study, we used MIMIC-IV version 3.1 (10.13026/kpb9-mt58), MIMIC III version 1.4 (10.13026/C2XW26) and NWICU version 0.1.0 (10.13026/s84w-1829); for more information on MIMIC, please visit (https://mimic.mit.edu). For MIMIC and NWICU data access, researchers must register with PhysioNet and get approval for dataset access after appropriate training: (https://physionet.org).

## Abbreviations

AI: Artificial Intelligence
BIDMC: Beth Israel Deaconess Medical Center
CF: Collaborative Filtering
CPRD: Clinical Practice Research Datalink
DR: Distance-based Representation
DR-NCA: Distance-based Representation with Neighborhood Component Analysis
DR-RBA: Distance-based Representation with Relief-Based Attribute weighting
DR-RBF: Distance-based Representation with Radial Basis Function
EHR: Electronic Health Records
GroupKFold: Group K-Fold cross-validation
ICD: International Classification of Diseases
ICD9: International Classification of Diseases version 9
ICD10: International Classification of Diseases version 10
ICU: Intensive Care Unit
IL: Illinois
ITR: Individual Treatment Rule
K: Number of neighbours (hyperparameter in similarity algorithms)
L: Total length of stay (normalised)
MA: Massachusetts
MAP@3: Mean Average Precision at position three
MIMIC-III: Medical Information Mart for Intensive Care III
MIMIC-IV: Medical Information Mart for Intensive Care IV
NCA: Neighborhood Component Analysis
NHS: National Health Service
NWICU: Northwestern Intensive Care Unit
P: Penalty for switching medications
RBF: Radial Basis Function
ReliefF: Relief-Based Feature selection algorithm
RMSE: Root-Mean-Square Error
S: Time between medication switches (normalised)
SD: Standard Deviation
SQL: Structured Query Language
T: Time between admissions (normalised)
UK: United Kingdom
USA: United States of America
V: Number of visits (normalised)
ε: Small value to prevent division by zero

## References

[1] Huhn M, et al. Comparative efficacy and tolerability of 32 oral antipsychotics for the acute treatment of adults with multi-episode schizophrenia: a systematic review and network meta-analysis. Lancet. 2019;394:939–51. 31303314 10.1016/S0140-6736(19)31135-3 PMC6891890

[2] Schneider-Thoma J, et al. Comparative efficacy and tolerability of 32 oral and long-acting injectable antipsychotics for the maintenance treatment of adults with schizophrenia: a systematic review and network meta-analysis. Lancet. 2022;399:824–36. 35219395 10.1016/S0140-6736(21)01997-8

[3] Leucht S, et al. Comparative efficacy and tolerability of 15 antipsychotic drugs in schizophrenia: a multiple-treatments meta-analysis. Lancet. 2013;382:951–62. 23810019 10.1016/S0140-6736(13)60733-3

[4] O’Donoghue B, Piacenza F, Plapp H, Siskind D, Lyne J. Response rates to sequential trials of antipsychotic medications according to algorithms or treatment guidelines in psychotic disorders. A systematic review and meta-analysis. Schizophr Res. 2024;268:193–204. 38493023 10.1016/j.schres.2024.02.035

[5] Lally J, MacCabe JH. Antipsychotic medication in schizophrenia: a review. Br Med Bull. 2015;114:169–79. 25957394 10.1093/bmb/ldv017

[6] Koutsouleris N, et al. Multisite prediction of 4-week and 52-week treatment outcomes in patients with first-episode psychosis: a machine learning approach. Lancet Psychiatry. 2016;3:935–46. 27569526 10.1016/S2215-0366(16)30171-7

[7] Passos IC, Mwangi B, Kapczinski F. Big data analytics and machine learning: 2015 and beyond. Lancet Psychiatry. 2016;3:13–5. 26772057 10.1016/S2215-0366(15)00549-0

[8] Del Fabro L, et al. Machine learning methods to predict outcomes of Pharmacological treatment in psychosis. Transl Psychiatry. 2023;13:75. 36864017 10.1038/s41398-023-02371-z PMC9981732

[9] Tay JL, Htun KK, Sim K. Prediction of clinical outcomes in psychotic disorders using artificial intelligence methods: A scoping review. Brain Sci. 2024;14:878. 39335374 10.3390/brainsci14090878 PMC11430394

[10] Etemadi M, et al. A systematic review of healthcare recommender systems: open issues, challenges, and techniques. Expert Syst Appl. 2023;213:118823.

[11] De Croon R, et al. Health recommender systems: systematic review. J Med Internet Res. 2021;23:e18035. 34185014 10.2196/18035 PMC8278303

[12] Tran TNT, Felfernig A, Trattner C, Holzinger A. Recommender systems in the healthcare domain: state-of-the-art and research issues. J Intell Inf Syst. 2021;57:171–201.

[13] Graf L et al. Acceptance of a digital therapy recommender system for psoriasis. BMC Med Inf Decis Mak. 2023;23. 10.1186/s12911-023-02246-9 PMC10401871 37542251

[14] Mai A, Voigt K, Schübel J, Gräßer F. A drug recommender system for the treatment of hypertension. BMC Med Inf Decis Mak. 2023;23. 10.1186/s12911-023-02170-y PMC10170737 37161441

[15] Gräßer F, et al. A pharmaceutical therapy recommender system enabling shared decision-making. User Model User-adapt Interact. 2022;32:1019–62.

[16] Raza S, et al. A comprehensive review of recommender systems: transitioning from theory to practice. 2024.

[17] Nagomiya S, Sanjay S, Shriram Kumar AN. Personalized recommender system for e-commerce. In: 2nd International Conference on Emerging Research in Computational Science (ICERCS 2024). Institute of Electrical and Electronics Engineers Inc.; 2024. 10.1109/ICERCS63125.2024.10895315

[18] Prabakaran R, Pradeepkandhasamy J, Arun MA. Survey on recommendation systems using collaborative filtering techniques. In: Proceedings of the 5th International Conference on Smart Systems and Inventive Technology (ICSSIT 2023). Institute of Electrical and Electronics Engineers Inc.; 2023. p. 1445–1450. 10.1109/ICSSIT55814.2023.10060889

[19] Padhy SK, Singh AK, Vetrivelan P. Item-based collaborative filtering blockchain for secure movie recommendation system. 2022;937–48. 10.1007/978-981-16-4625-6_93

[20] Wu C-S, et al. Development and validation of a machine learning individualized treatment rule in First-Episode schizophrenia. JAMA Netw Open. 2020;3:e1921660. 32083693 10.1001/jamanetworkopen.2019.21660 PMC7043195

[21] Bazargan-Hejazi S, et al. Examining racial disparity in psychotic disorders related ambulatory care visits: an observational study using national ambulatory medical care survey 2010–2015. BMC Psychiatry, 2023;23. 10.1186/s12888-023-05095-y PMC10436449 37592201

[22] Schwartz RC. Racial disparities in psychotic disorder diagnosis: A review of empirical literature. World J Psychiatry. 2014;4:133. 25540728 10.5498/wjp.v4.i4.133 PMC4274585

[23] Johnson AEW, et al. MIMIC-IV, a freely accessible electronic health record dataset. Sci Data. 2023;10:1. 36596836 10.1038/s41597-022-01899-x PMC9810617

[24] Johnson AEW, et al. MIMIC-III, a freely accessible critical care database. Sci Data. 2016;3:160035. 27219127 10.1038/sdata.2016.35 PMC4878278

[25] Moukheiber D, et al. Northwestern ICU (NWICU) database. PhysioNet. 2024. RRID:SCR_007345. Preprint.

[26] Johnson A, Pollard T, Mark R. MIMIC-III clinical database CareVue subset. 2022. Dataset preprint.

