# Supplementary material for: Developing and validating a machine learning pharmaceutical therapy recommender system for US-based hospital in-patients with schizophrenia spectrum disorders
Source: BMC Psychiatry. 2025 Dec 1;26:10. doi: 10.1186/s12888-025-07657-8 (PMC12777454; doi:10.1186/s12888-025-07657-8)
Supplement: Supplementary file 1 — Supplementary Material 1 [file 12888_2025_7657_MOESM1_ESM.docx]

| **Supplementary Table 1** | | | | | |
| --- | --- | --- | --- | --- | --- |
| **Visit** | **Affinity Score Formulation** | **RMSE (±)** | **MAP@3 (±)** | **Overlap (±)** | **Best K (±)** |
| **1** | Original (equal) | 0.117 ± 0.007 | 0.702 ± 0.019 | 0.874 ± 0.023 | 7.0 ± 4.0 |
|  | Stability-heavy | 0.133 ± 0.008 | 0.811 ± 0.030 | 0.870 ± 0.022 | 6.0 ± 2.0 |
|  | Patient-burden | 0.136 ± 0.008 | 0.821 ± 0.029 | 0.870 ± 0.022 | 6.0 ± 2.0 |
| **2** | Original (equal) | 0.138 ± 0.013 | 0.297 ± 0.056 | 0.662 ± 0.051 | 7.0 ± 4.0 |
|  | Stability-heavy | 0.159 ± 0.021 | 0.374 ± 0.050 | 0.672 ± 0.051 | 6.0 ± 2.0 |
|  | Patient-burden | 0.158 ± 0.021 | 0.360 ± 0.050 | 0.670 ± 0.051 | 6.0 ± 2.0 |
| **3** | Original (equal) | 0.094 ± 0.008 | 0.012 ± 0.010 | 0.844 ± 0.040 | 7.0 ± 4.0 |
|  | Stability-heavy | 0.121 ± 0.010 | 0.012 ± 0.010 | 0.840 ± 0.045 | 6.0 ± 2.0 |
|  | Patient-burden | 0.122 ± 0.010 | 0.012 ± 0.010 | 0.839 ± 0.045 | 6.0 ± 2.0 |
| **All** | Original (equal) | 0.118 ± 0.004 | 0.493 ± 0.021 | 0.814 ± 0.020 | 7.0 ± 4.0 |
|  | Stability-heavy | 0.138 ± 0.005 | 0.602 ± 0.020 | 0.814 ± 0.018 | 6.0 ± 2.0 |
|  | Patient-burden | 0.139 ± 0.005 | 0.597 ± 0.019 | 0.813 ± 0.018 | 6.0 ± 2.0 |
